# Circulating interleukin-8 and osteopontin are promising biomarkers of clinical outcomes in advanced melanoma patients treated with targeted therapy

**DOI:** 10.1186/s13046-024-03151-3

**Published:** 2024-08-15

**Authors:** Lauretta Levati, Claudio Tabolacci, Antonio Facchiano, Francesco Facchiano, Ester Alvino, Gian Carlo Antonini Cappellini, Enrico Scala, Laura Bonmassar, Simona Caporali, Pedro Miguel Lacal, Antonella Bresin, Federica De Galitiis, Giandomenico Russo, Stefania D’Atri

**Affiliations:** 1 Laboratory of Molecular Oncology, Istituto Dermopatico Dell’Immacolata, IDI-IRCCS, Via Dei Monti Di Creta 104, 00167 Rome, Italy; 2 Department of Oncology and Molecular Medicine, Istituto Superiore Di Sanità, Viale Regina Elena 299, 00161 Rome, Italy; 3 Institute of Translational Pharmacology, National Council of Research, Via Fosso del Cavaliere 100, 00133 Rome, Italy; 4 Department of Oncology and Dermatological Oncology, Istituto Dermopatico Dell’Immacolata, IDI-IRCCS, Via Dei Monti Di Creta 104, 00167 Rome, Italy; 5 Clinical and Laboratory Molecular Allergy Unit, Istituto Dermopatico Dell’Immacolata, IDI-IRCCS, Via Dei Monti Di Creta 104, 00167 Rome, Italy; 6 Present Address: Research Coordination and Support Service, Istituto Superiore Di Sanità, Viale Regina Elena 299, 00161 Rome, Italy; 7 Present Address: UOC Oncologia, Interpresidio ASL RM2, Via Dei Monti Tiburtini 387, 00157 Rome, Italy; 8 Present Address: Regional Transplant Center Lazio (CRTL), San Camillo Hospital, Circonvallazione Gianicolense 87, 00152 Rome, Italy

**Keywords:** Melanoma, Targeted therapy, Cytokines, IL-8, Osteopontin, BDNF, Progression-free survival, Overall survival

## Abstract

**Background:**

Circulating cytokines can represent non-invasive biomarkers to improve prediction of clinical outcomes of cancer patients. Here, plasma levels of IL-8, CCL4, osteopontin, LIF and BDNF were determined at baseline (T0), after 2 months of therapy (T2) and, when feasible, at progression (TP), in 70 melanoma patients treated with BRAF and MEK inhibitors. The association of baseline cytokine levels with clinical response, progression-free survival (PFS) and overall survival (OS) was evaluated.

**Methods:**

Cytokine concentrations were measured using the xMAP technology. Their ability to discriminate between responding (Rs) and non-responding (NRs) patients was assessed by Receiver Operating Characteristics analysis. PFS and OS were estimated with the Kaplan–Meier method. The Cox proportional hazard model was used in the univariate and multivariate analyses to estimate crude and adjusted hazard ratios with 95% confidence intervals.

**Results:**

CCL4 and LIF were undetectable in the majority of samples**.** The median osteopontin concentration at T0 and T2 was significantly higher in NRs than in Rs. The median T0 and T2 values of IL-8 were also higher in NRs than in Rs, although the statistical significance was not reached. No differences were detected for BDNF. In 39 Rs with matched T0, T2, and TP samples, osteopontin and IL-8 significantly decreased from T0 to T2 and rose again at TP, while BDNF levels remained unchanged. In NRs, none of the cytokines showed a significant decrease at T2. Only osteopontin demonstrated a good ability to discriminate between Rs and NRs. A high IL-8 T0 level was associated with significantly shorter PFS and OS and higher risk of progression and mortality, and remained an independent negative prognostic factor for OS in multivariate analysis. An elevated osteopontin T0 concentration was also significantly associated with worse OS and increased risk of death. Patients with high IL-8 and high osteopontin showed the lowest PFS and OS, and in multivariate analysis this cytokine combination remained independently associated with a three- to six-fold increased risk of mortality.

**Conclusion:**

Circulating IL-8 and osteopontin appear useful biomarkers to refine prognosis evaluation of patients undergoing targeted therapy, and deserve attention as potential targets to improve its clinical efficacy.

**Supplementary Information:**

The online version contains supplementary material available at 10.1186/s13046-024-03151-3.

## Background

During the last decade, remarkable progress has been achieved in the treatment of cutaneous melanoma, an aggressive tumor whose incidence is constantly increasing worldwide [1].

In the metastatic setting, targeted therapy with BRAF and MEK inhibitors (BRAFi, MEKi) approved for BRAF-mutant tumors, and immunotherapy with inhibitors of immune checkpoints, approved for both BRAF-mutant and BRAF wild-type melanomas, have significantly increased objective response rate (ORR), progression-free survival (PFS) and overall survival (OS) of patients with respect to dacarbazine chemotherapy [2, 3]. The combination of BRAFi + MEKi, representing the standard-of-care for BRAF targeted approaches, produces ORR of 65–75% and PFS and OS rates of 15–22% and 30–38%, respectively, at 5 years [4–6]. Monotherapy with anti-CTLA-4 (ipilimumab) and anti-PD1 (nivolumab and pembrolizumab) monoclonal antibodies, results in ORR of 10–15% and 30–40%, respectively, along with 5-year PFS and OS rates of 8–10% and 20–25% for ipilimumab, and 20–30% and 40–45% for nivolumab and pembrolizumab [7, 8]. Improved clinical outcomes have been reported for the combination of nivolumab and ipilimumab, with a 5-year PFS and OS rate of 36% and 52%, respectively, and an ORR of 58% [7]. Recently, the association of nivolumab plus relatlimab, a monoclonal antibody targeting LAG-3 [9], has been approved for first-line treatment of unresectable or metastatic melanomas with PD-L1 expression lower than 1%, based on superior outcomes in terms of ORR, PFS and OS compared to nivolumab monotherapy [10].

Although the new available drugs have significantly improved the prognosis of metastatic melanoma, the percentage of patients showing primary resistance to therapy, especially to immune checkpoint inhibitors, is still relevant [11, 12]. Furthermore, the development of secondary resistance, particularly against BRAFi and MEKi, limits the long-term efficacy of the therapy [13, 14]. Consequently, there is still a need to identify novel prognostic indicators and predictors of clinical response, as well as to develop novel therapeutic approaches.

Cytokines are a class of secreted signaling molecules comprising growth factors, chemokines, interleukins, interferons, tumor necrosis factors, angiogenic and antiangiogenic factors [15, 16]. Cytokines and their receptors are expressed by a variety of immune and nonimmune cells and orchestrate a plethora of physiological processes, including inflammation, angiogenesis, innate and acquired immunity, cell migration, differentiation, proliferation and apoptosis [15, 16]. Perturbations in cytokine signaling, due to aberrant expression of the cytokines themselves and/or their receptors, have been implicated in tumor development and progression, immune evasion and metastasis [15, 16]. Moreover, cytokines secreted by tumor cells and/or tumor-associated stromal cells have emerged as pivotal determinants of therapeutic resistance [17, 18]. Consequently, cytokines and their receptors hold promise as diagnostic and prognostic biomarkers and predictors of patients’ response to therapy, as well as therapeutic targets.

Recent years have seen an increase in the validation of peripheral blood as a "liquid biopsy" capable of providing invaluable insights into the molecular landscape of a patient's tumor through the analysis of circulating DNA, RNA, microRNAs, and proteins, often employing high-throughput methodologies [19–22]. In this context, differential expression of serum/plasma cytokines between patients and healthy controls has been reported in a variety of cancers [23], underscoring the utility of cytokines as discriminating indicators. Moreover, several investigations have identified specific circulating cytokines or cytokine signatures, as being associated with patients’ PFS, OS, and/or response to therapy [17, 18, 23].

Melanoma cells can express a variety of cytokines that acting in a paracrine fashion, modulate the tumor microenvironment to promote angiogenesis, immunosuppression, tumor growth and metastasis [24–26]. Additionally, melanoma cells frequently express specific cytokines along with their cognate receptors, thereby activating autocrine loops of proliferation and invasion [25]. On the other hand, cytokines released by major tumor-associated stromal cells (e.g. endothelial cells, fibroblasts, macrophages, lymphocytes) can also sustain melanoma cell survival, proliferation and invasion and confer resistance to antitumor agents [25, 27].

Circulating levels of several cytokines have been found to be altered in melanoma patients as compared with healthy subjects and to be correlated with tumor clinicopathological features and/or patients’ prognosis [28–36]. In addition, various investigations have shown the potential role of selected circulating cytokines as predictors of clinical outcomes in advanced melanoma patients subjected to immunotherapy [37–40] or BRAFi and MEKi [41–44].

In the present investigation, plasma levels of interleukin-8 (IL-8), C–C motif chemokine ligand 4 (CCL4), osteopontin, leukemia inhibitory factor (LIF) and brain derived neurotrophic factor (BDNF), were determined at baseline, after 2 months of treatment and, when feasible, at progression, in a cohort of 70 stage III-IV melanoma patients subjected to BRAFi monotherapy or the combination of BRAFi and MEKi and their association with clinical response, PFS and OS was evaluated.

IL-8 and CCL4 were selected based on previous findings showing that their circulating levels could predict response to therapy and/or OS of melanoma patients treated with immunotherapy [37, 38, 40, 43] or targeted therapy [42, 43]. Osteopontin, BDNF and LIF were selected because they have been implicated in melanoma growth, progression and metastasis [45–50], shown to be detectable in serum/plasma of patients with melanoma [29–33, 36], and identified as potential circulating biomarkers of response to therapy and/or PFS or OS across various malignancies [49–56]. However, investigations exploring their associations with clinical outcomes in melanoma patients undergoing BRAFi and MEKi regimens are currently lacking.

## Materials and methods

### Study population

A total of 70 patients diagnosed with inoperable stage IIIC/IIID or stage IV melanoma, consecutively enrolled at IDI-IRCCS from 2013 to 2019 and treated with either dabrafenib or vemurafenib monotherapy, or initially with dabrafenib for a period of 4–8 months and then with dabrafenib plus trametinib, or with the association of dabrafenib + trametinib or vemurafenib + cobimetinib, and for whom peripheral blood samples had been collected before the start of therapy (T0), after two months of treatment (T2) and, when feasible, at disease progression (TP), were included in the study. Demographic and clinicopathological features of patients are illustrated in Table 1.

**Table 1:** Baseline demographic and clinical characteristics of melanoma patients included in the study

**Number of Pts**	70
**Stage**^**a**^	
**IIIC**	7
**IIID**	1
**IV**	62
M1a	13
M1b	12
M1c	21
M1d	16
**Male**	45
**Female**	25
**Age (years, range)**	19–85
**sLDH**	
Normal	43
High^b^	27
**ECOG PS**^**c**^	
0	38
1	20
2	11
3	1
**Previous Therapy**	
Yes	7
No	63
**Targeted Therapy**^**d**^	
DAB	6
VEM	9
DAB--->DAB + TRAM	7
DAB + TRAM	43
VEM + COBI	5
**Best Response**^**e**^	
CR	17
PR	42
SD	4^f^
PD	7

^a^Disease stage before the beginning of targeted therapy according to 8th Edition of the AJCC *Cancer Staging Manual.* ^b^ > 1.5 upper limit of normal values. ^c^Eastern Cooperative Oncology Group (ECOG) Performance Status. ^d^*DAB *dabrafenib, *TRAM *trametinib, *VEM *vemurafenib, *COBI *cobimetinib. ^e^*CR *complete response, *PR *partial response, *SD *stable disease, *PD *progressive disease, according to RECIST 1.1 criteria. ^f^One patients with SD > 2 years was included in the group of responders

Dabrafenib was administered at the dose of 150 mg BID, vemurafenib at the dose of 960 mg BID, dabrafenib + trametinib at the dose of 150 mg BID and 2 mg/die, respectively, and vemurafenib + cobimetinib at the dose of 960 mg BID and 60 mg/die, respectively, for three weeks followed by a one-week break.

Baseline assessments included medical history review, physical examination, evaluation of biochemical parameters and radiologic tumor assessment with computer tomography (CT) or positron emission tomography scans. Monthly follow-up included physical examination and biochemical parameter evaluations, while tumor response was assessed with CT every three months, or more frequently if deemed necessary. Tumor response was classified as complete response (CR), partial response (PR), stable disease (SD) or progressive disease (PD) according to RECIST 1.1 criteria. Patients achieving CR or PR were classified as “Responders” (Rs), whereas patients with SD or PD as best response were classified as “Non-responders” (NRs) with the exception of one patient who experienced a SD for more than 2 years and was included in the group of Rs. PFS was defined as the time from the start of therapy to the first observation of disease progression per RECIST 1.1 or death (event) or last follow-up (censored). OS was calculated from the start of therapy to death (event) or last follow-up (censored). Median follow-up was 76.19 months.

The study was conducted in accordance with Good Clinical Practice Guidelines and the principles outlined in the Declaration of Helsinki. The study was also approved by the IDI-IRCCS Ethics Committee (ID #407/1, 2013; ID #407/3, 2021) and a written informed consent was obtained from all patients.

### Plasma preparation and cytokine quantification

Blood samples were collected into BD Vacutainer® tubes (#367,704, BD Biosciences, Plymouth, UK) and double centrifuged at 1200 × *g* for 10 min at 4 °C. The resultant plasma was aliquoted and stored at − 80 °C until use.

Quantification of osteopontin, IL-8, CCL4, BDNF, and LIF concentrations in plasma samples was performed using xMAP technology and a customized assay (Human Luminex® Discovery Assay, cat. #LXSAHM, R&D Systems, Minneapolis, MN). Secretome profiling was performed according to the manufacturer’s instructions with slight modifications. Briefly, plasma samples were diluted (1:1 vol/vol) with assay buffer. After incubation with magnetic beads (overnight at 4 °C), samples were incubated with detection antibodies (1 h at room temperature). Streptavidin-PE solution was then added (30 min at room temperature). Measures were executed in duplicate on a Luminex Bio-Plex 200 workstation equipped with magnetic washer (Bio-Rad, Hercules, CA) and analyzed using Bio-Plex Software Manager™ 6.1 (Bio-Rad). An eight-point standard dilution series was prepared and a 5PL curve for each cytokine was generated by the software. Cytokine concentration was expressed in pg/ml.

### Statistical analysis

Plasma levels of the studied cytokine are presented as median and Interquartile Range (IQR). The Mann–Whitney U test was used to compare between-group differences (i.e. NRs *versus* Rs) while the Wilcoxon matched-pairs signed-rank test was used to evaluate before–after differences (i.e. T2 *vs* T0, TP *vs* T0, TP *vs* T2).

Receiver Operating Characteristics (ROC) curves were generated to assess the ability of T0 cytokine concentrations to discriminate between Rs and NRs.

Differences in T0 cytokine levels according to patients’ disease stage were assessed by the Kruskal–Wallis test followed by the post-hoc Dunn’s test for multiple comparisons.

PFS and OS curves were estimated with the Kaplan–Meier method. The log-rank test was used to compare the PFS and OS curves among patient groups defined on the basis of the T0 median value of each cytokine determined in the entire cohort, as well as of cytokine combinations.

The Cox proportional hazard model was used in the univariate and multivariate analyses to estimate crude and adjusted hazard ratios (HR) with corresponding 95% confidence intervals (CIs).

For all comparisons, a two-tailed *p* value < 0.05 was considered to be statistically significant.

Statistical analyses were performed using the GraphPad Prism software version 9.3.0 (Boston, MA) and the IBM SPSS Statistics 29.0 software (Chicago, IL).

## Results

### Cytokine expression in plasma samples

Plasma levels of osteopontin, IL-8, BDNF, CCL4, and LIF were determined in a cohort of 70 patients treated with either vemurafenib or dabrafenib monotherapy, with dabrafenib for 4–8 months and then with dabrafenib + trametinib, or with the combination of dabrafenib + trametinib or vemurafenib + cobimetinib. Sixty patients were classified as Rs and 10 patients as NRs. In the NR group, cytokine quantification was performed in plasma samples collected at baseline (T0) and after two months of therapy (T2), while in the R group cytokine levels were determined also in samples obtained at progression (TP) whenever available. At the time of cytokine assessment, 9 Rs were still in response and no matched TP samples were therefore available. Moreover, the TP sample was unavailable for 5 Rs who had been lost at follow-up (4 cases) or had died from causes other than melanoma before experiencing progression (1 case), and for 7 Rs who had progressed. Overall, 70 T0 and T2 samples and 39 TP samples were subjected to analysis.

LIF and CCL4 were undetectable in all and in the majority of samples, respectively. Only osteopontin, IL-8 and BDNF determinations were, therefore, subjected to the subsequent analyses.

**Fig. 1:**
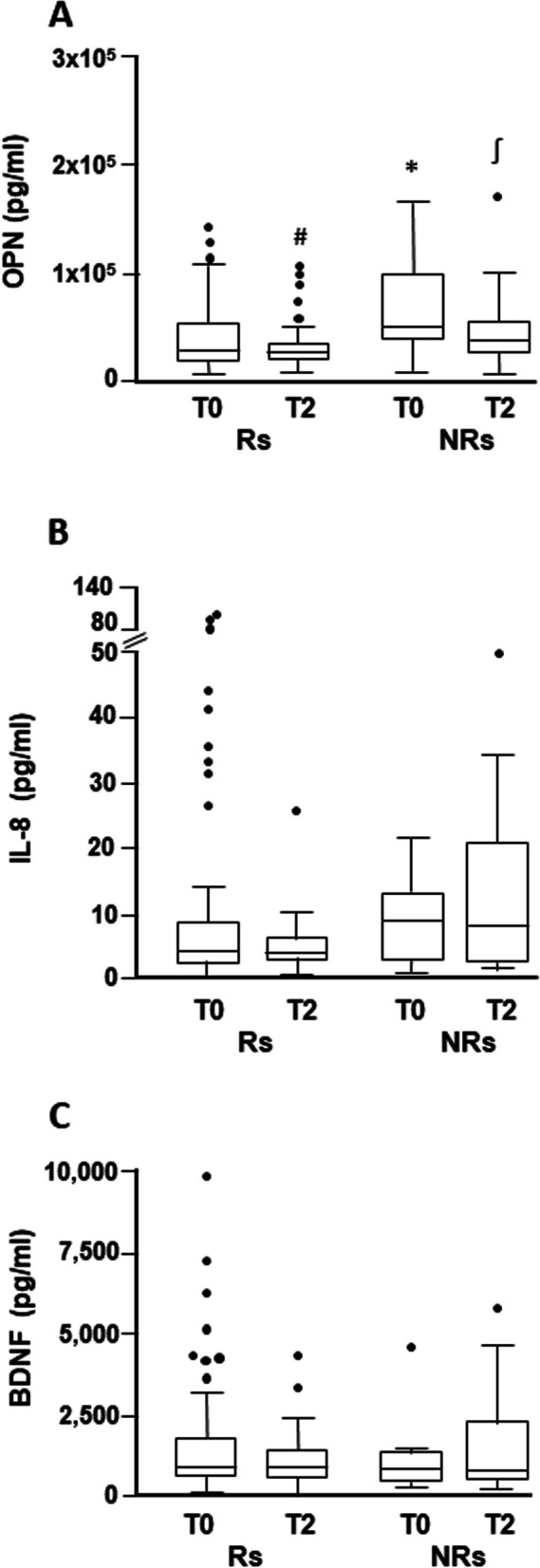
Box-and-whisker diagrams of cytokine T0 and T2 plasma levels in melanoma patients treated with targeted therapy. Osteopontin (OPN) (**A**), IL-8 (**B**) and BDNF (**C**) levels were measured by xMAP technology in plasma samples obtained from 70 patients, comprising 60 responders (Rs) and 10 non-responders (NRs), before treatment initiation (T0), and after two months of treatment (T2). The edges of each box represent the 75th and 25th percentile, respectively, and whiskers are defined according to Tukey method. The horizontal bar within each box indicates the median. The outliers are reported as dots. Data were analyzed by nonparametric Mann–Whitney U test to compare differences between groups, and by the Wilcoxon matched-pairs signed-rank test for the before-after differences. *p = 0.024 comparing T0/NRs *vs* T0/Rs. ^∫^*p* = 0.044 comparing T2/NRs *vs* T2/Rs. #*p* = 0.011 comparing T2/Rs *vs* T0/Rs

Statistically significant differences were observed in osteopontin levels between the Rs and NRs groups at both T0 and T2 (Fig. 1A). At T0, the median value of osteopontin was 29,629 (IQR 19,779–55,483) pg/ml for Rs and 52,651 (IQR 40,957–101,963) pg/ml for NRs (*p* = 0.024). At T2, the median value of the protein was 27,388 (IQR 20,330–36,027) pg/ml for Rs and 37,902 (IQR 26,922–58,435) pg/ml for NRs (*p* = 0.044). The difference in osteopontin concentration observed between T2 and T0 samples (all matched) was statistically significant for the group of Rs (*p* = 0.011) but not for the group of NRs (*p* = 0.232). Regarding IL-8, a higher median value was observed in the NR group compared to the R group at both T0 (8.89 pg/ml, IQR 2.80–13.25 *vs* 4.14 pg/ml, IQR 2.48–8.86) and T2 (8.18 pg/ml, IQR 2.62–21.02 *vs* 3.89 pg/ml, IQR 2.84–6.35), although the statistical significance was not reached. Moreover, no significant differences were observed between T0 and T2 samples in either group (Fig. 1B). Comparable median values of BDNF were detected in NRs and Rs at both T0 (871.6 pg/ml, IQR 446.9–1,401 *vs* 920.7 pg/ml, IQR 666.5–1,848) and T2 (859.4 pg/ml, IQR 534.4–2,348 *vs* 932.9 pg/ml, IQR 574.3–1,458), with no significant variation between the two time points in each group (Fig. 1C).

**Fig. 2:**
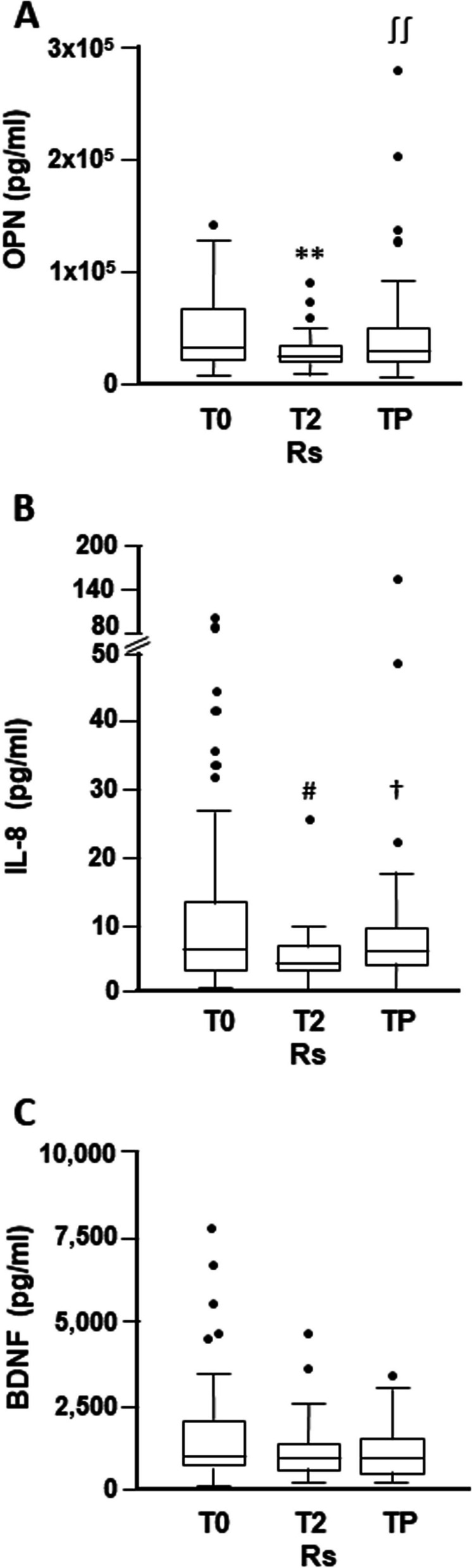
Box-and-whisker diagrams of cytokine T0, T2 and TP plasma levels in melanoma patients responding to targeted therapy. Osteopontin (OPN) (**A**), IL-8 (**B**) and BDNF (**C**) levels were measured by xMAP technology in matched plasma samples obtained from 39 responders (Rs) before treatment initiation (T0), after two months of treatment (T2), and at disease progression (TP). The edges of each box represent the 75th and 25th percentile, respectively, and whiskers are defined according to Tukey method. The horizontal bar within each box indicates the median. The outliers are reported as dots. Data were analyzed by the Wilcoxon matched-pairs signed-rank test. **p = 0.0002 comparing T2 *vs* T0 and ^∫∫^p = 0.006 comparing TP *vs* T2 for osteopontin. #*p* = 0.040 comparing T2 *vs* T0 and.^†^*p* = 0.046 comparing TP *vs* T2 for IL-8

Among the Rs, thirty-nine patients had also a TP sample available. In this more restricted cohort of patients, we therefore compared the levels of the three cytokines at the different time points investigated. As illustrated in Fig. 2A, the results confirmed the significant decrease of osteopontin median concentration at T2 with respect to T0 (27,213 pg/ml, IQR 21,065–36,070, *vs* 33,679 pg/ml, IQR 22,273–69,221) (*p* = 0.0002). Moreover, the TP median value (30,928 pg/ml, IQR 20,563–52,085) was significantly higher than that observed at T2 (*p* = 0.006) and comparable to that determined at T0. Similarly, for IL-8, data analysis evidenced a significant reduction in the cytokine median value at T2 (4.04 pg/ml, IQR 2.92–6.95) with respect to T0 (6.17 pg/ml, IQR 2.83–13.54) (*p* = 0.040), and a significant enhancement in its concentration from T2 to TP (5.81 pg/ml, IQR 3.9–9.7) (*p* = 0.046), but not substantial differences between T0 and TP samples (Fig. 2B). As observed for the entire cohort of Rs, BDNF median value did not change from T0 to T2 (943.3 pg/ml, IQR 718.6–1,963, *vs* 934.0, IQR 603.2–1,347). Moreover, the median cytokine concentration at TP (919.4 pg/ml, IQR 522.2–1,488) remained comparable to that of T0 and T2 (Fig. 2C).

We also investigated whether differences existed in T0 cytokine levels according to patients’ disease stage. We did not observe a clear trend in cytokine levels across the different stages. As illustrated in Supplementary Fig. 1, statistically significant differences were only observed between osteopontin levels of patients with stage M1c and patients with stage IIIC/IIID (*p* = 0.013) or stage M1d (*p* = 0.031), and between IL-8 levels of patients with stage M1c and patients with stage M1b (*p* = 0.005).

### Evaluation of the ability of osteopontin, IL-8 and BDNF T0 plasma levels to discriminate NRs from Rs and of their association with PFS and OS

T0 levels of the three cytokines under investigation were initially subjected to ROC analysis to assess their potential as predictive biomarkers of patients’ response to therapy. We considered each cytokine individually, as well as all the possible cytokine combinations. The median T0 concentrations of the cytokines determined in the entire patient cohort were selected as cut-off values. As shown in Table 2, a good ability to discriminate between Rs and NRs was observed for osteopontin (AUC = 0.72, *p* = 0.026). T0 levels above 33,687.82 pg/ml were able to predict unresponsiveness to therapy with a sensitivity of 57% and a specificity of 90%. Conversely, IL-8 and BDNF exhibited a poor predictive performance (Table 2). None of the tested cytokine combinations yielded an AUC value surpassing that of osteopontin alone (data not shown).

**Table 2:** AUC for T0 plasma levels of osteopontin, IL-8 and BDNF

Cytokine	AUC (95% CI)^a^	*P*	Cut-off^b^	Sensitivity (%)	Specificity (%)
Osteopontin	0.72 (0.54–0.90)	0.026	33,687.82	57	90
IL-8	0.56 (0.36–0.76)	0.520	4.32	52	60
BDNF	0.58 (0.38–0.77)	0.420	920.68	50	50

^a^*AUC *area under curve, *CI *confidence interval

^b^Median value in pg/ml for to the entire cohort of 70 patients

We next assessed the association of T0 levels of the three cytokines with PFS and OS. To this end, the median concentration of each cytokine was used to categorize patients into those with high (i.e. > median value) or low (i.e. ≤ median value) level of the cytokine.

**Fig. 3:**
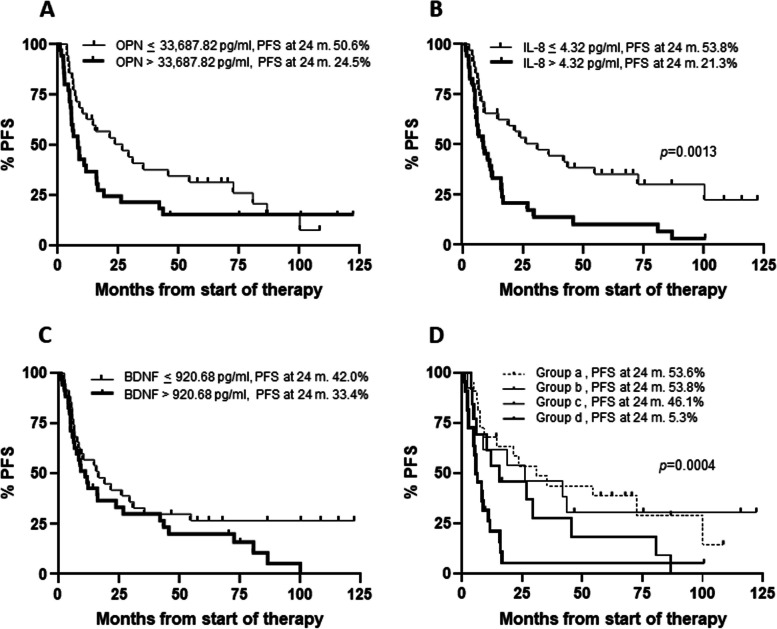
Kaplan–Meier curves for progression-free survival (PFS) of melanoma patients treated with targeted therapy. Patients (n. 70) were categorized based on their T0 levels of osteopontin (OPN) (**A**), IL-8 (**B**), BDNF(**C**), osteopontin and IL-8 combinations (**D**). Group (a): osteopontin ≤ 33,687.82 pg/ml and IL-8 ≤ 4.32 pg/ml; group (b): osteopontin > 33,687.82 pg/ml and IL-8 ≤ 4.32 pg/ml; group (c): osteopontin ≤ 33,687.82 pg/ml and IL-8 > 4.32 pg/ml; group (d): osteopontin > 33,687.82 pg/ml and IL-8 > 4.32 pg/ml. *p*-values are from log-rank test (**A**, **B**, **C**) and log-rank test for trend (**D**). The estimated percentages of PFS at 24 months from the start of therapy are also reported for each comparison

 Median PFS of all 70 patients was 12.1 months. Patients with osteopontin ≤ 33,687.82 pg/ml showed a median PFS of 26.7 months, whereas patients with osteopontin > 33,687.82 pg/ml had a median PFS of 8.3 months. However, the difference did not reach the statistical significance (Fig. 3A). PFS was significantly longer in patients with IL-8 ≤ 4.32 pg/ml, compared to those with IL-8 > 4.32 pg/ml (31.0 *vs* 8.3 months, *p* = 0.0013) (Fig. 3B). As in the case of osteopontin, patients with elevated levels of BDNF (i.e. > 920.68 pg/ml) showed a shorter PFS than patients with low levels of the cytokine (i.e. ≤ 920.68 pg/ml) (10.9 *vs* 16.6 months), although the difference was not significant (Fig. 3C).

We then considered the combinations of the two best performing cytokines, namely osteopontin and IL-8. Patients were classified in four groups according to T0 levels of the two cytokines: group (a), comprising patients with low osteopontin and low IL-8; group (b), including patients with high osteopontin and low IL-8; group (c), consisting of patients with low osteopontin and high IL-8; group (d), including patients with high osteopontin and high IL-8. The estimated PFS was 31.0, 26.1, 15.6 and 6.0 months for group (a), (b), (c) and (d), respectively (log-rank test for trend, *p* = 0.0004) (Fig. 3D).

For each group of patients, the percentage of those without progression 24 months after the start of therapy was also evaluated and is shown in Fig. 3. Notably, only 5.3% of patients with unfavorable levels of both osteopontin and IL-8 were still in response at that time, whereas 53.6% of those with low osteopontin and IL-8 were still free of progression (Fig. 3D).

**Fig. 4:**
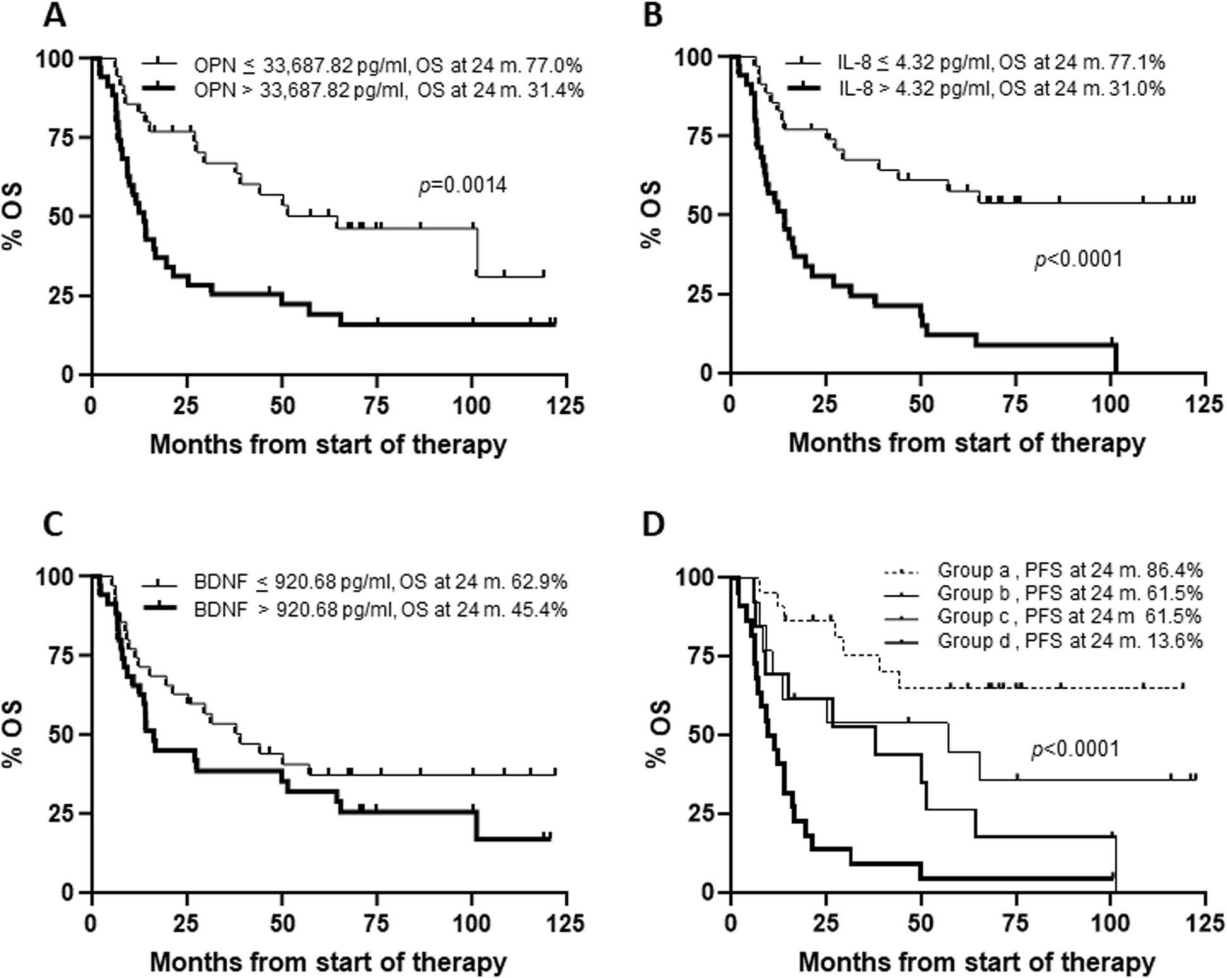
Kaplan–Meier curves for overall survival (OS) of melanoma patients treated with targeted therapy. Patients (n. 70) were categorized based on their T0 levels of osteopontin (**A**), IL-8 (**B**), BDNF(**C**), osteopontin and IL-8 combinations (**D**). Group (a): osteopontin ≤ 33,687.82 pg/ml and IL-8 ≤ 4.32 pg/ml; group (b): osteopontin > 33,687.82 pg/ml and IL-8 ≤ 4.32 pg/ml; group (c): osteopontin ≤ 33,687.82 pg/ml and IL-8 > 4.32 pg/ml; group (d): osteopontin > 33,687.82 pg/ml and IL-8 > 4.32 pg/ml. *p*-values are from log-rank test (**A**, **B**, **C**) and log-rank test for trend (**D**). The estimated percentages of OS at 24 months from the start of therapy are also reported for each comparison

Median OS for all patients was 27.5 months. Patients with high osteopontin exhibited a median OS of 13.5 months, whereas those with low osteopontin showed a median OS of 64.5 months (*p* = 0.0014) (Fig. 4A). A poorer survival outcome was also observed for patients with high IL-8 levels compared to those with low levels of the cytokine (13.9 months *vs* not reached, *p* < 0.0001) (Fig. 4B), and for patients of groups (b) (57.2 months), (c) (37.9 months) and (d) (10.6 months) with respect to those of group (a) (not reached) (log-rank test for trend, *p* < 0.0001) (Fig. 4D). Patients with elevated levels of BDNF exhibited a worse median OS than those with low levels of the cytokine (16.1 *v*s 39.2 months), although the difference was not statistically significant (Fig. 4C). Eighty-six percent of patients with low osteopontin and IL-8 were alive at 24 months from the start of therapy, whereas only 13.6% of those with high levels of the two cytokines were alive (Fig. 4D).

Based on plasma levels, ROC analysis and Kaplan–Meier curves we excluded BDNF from further investigations, whereas univariate Cox regression analysis for PFS and OS was conducted for T0 levels of osteopontin and IL-8, the combinations of the two cytokines and for patient baseline characteristics known to have prognostic value, comprising age, sex, disease stage, LDH and Eastern Cooperative Oncology Group (ECOG) performance status (PS). Due to the limited number of patients in groups (b) and (c) (13 in each group) the two classes of patients were considered together for univariate analyses.

A significantly increased risk of progression was observed for patients with high levels of IL-8 compared to patients with low levels of this cytokine, as well as for patients of group (d) with respect to those of group (a) (Table 3). Moreover, elevated osteopontin, elevated IL-8, the (b + c) and (d) combinations of the two cytokines were all associated with increased risk of mortality (Table 4). Male gender, stage M1c/M1d, elevated LDH and ECOG PS ≥ 1 were associated with increased risk of both progression and mortality (Table 3 and Table 4).

**Table 3:** Univariate Cox regression analysis for disease progression according to T0 plasma levels of osteopontin, IL-8 and clinical features of all patients

Characteristics	Events^a^/Patients	HR^b^ (95% CI^b^)	*P*^b^
**Osteopontin pg/ml**			
≤ 33,687.82	27/35	1	
> 33,687.82	29/35	1.60 (0.94–2.71)	0.083
**IL-8 pg/ml**			
≤ 4.32	24/35	1	
> 4.32	32/35	2.38 (1.38–4.09)	0.002
**Osteopontin and IL-8 combinations**^**c**^			
Group a	15/22	1	
Group b + group c	21/26	1.37 (0.70–2.66)	0.353
Group d	20/22	3.31 (1.65–6.64)	0.001
**sLDH**			
Normal	30/43	1	
High^d^	26/27	5.93 (3.12–11.27)	< 0.0001
**Stage**^**e**^			
IIIC/D + M1a + M1b	23/33	1	
M1c + M1d	33/37	2.67 (1.56–4.63)	< 0.0001
**Gender**			
Female	17/25	1	
Male	39/45	1.99 (1.12–3.57)	0.019
**Age, years**			
≤ 60	32/37	1	
> 60	24/33	0.68 (0.40–1.16)	0.156
**ECOG PS**^**f**^			
0	28/38	1	
≥ 1	28/32	2.28 (1.33–3.88)	0.003

^a^Event: disease progression

^b^*HR *Hazard Ratio, *CI *Confidence Interval, *P *probability. Estimated by Cox's regression model

^c^Group (a): osteopontin ≤ 33,687.82 pg/ml and IL-8 ≤ 4.32 pg/ml; group (b): osteopontin > 33,687.82 pg/ml and IL-8 ≤ 4.32 pg/ml; group (c): osteopontin ≤ 33,687.82 pg/ml and IL-8 > 4.32 pg/ml; group (d): osteopontin > 33,687.82 pg/ml and IL-8 > 4.32 pg/ml

^d^ > 1.5 upper limit of normal values

^e^Disease stage before the beginning of targeted therapy according to 8th Edition of the AJCC *Cancer Staging Manual*

^f^ECOG PS, Eastern Cooperative Oncology Group Performance Status

**Table 4:** Univariate Cox regression analysis for mortality according to T0 plasma levels of osteopontin, IL-8 and clinical features of all patients

Characteristics	Events^a^/Patients	HR^b^ (95% CI^b^)	*P*^b^
**Osteopontin pg/ml**			
≤ 33,687.82	29/35	1	
> 33,687.82	18/35	2.54 (1.41–4.60)	0.002
**IL-8 pg/ml**			
≤ 4.32	32/35	1	
> 4.32	15/35	3.98 (2.13–7.45)	< 0.0001
**Osteopontin and IL-8 combinations**^**c**^			
Group a	7/22	1	
Group b + group c	19/26	2.89 (1.21–6.88)	0.017
Group d	21/22	8.20 (3.40–19.80)	< 0.0001
**sLDH**			
Normal	23/43	1	
High^d^	24/27	6.05 (3.10–11.79)	< 0.0001
**Stage**^**e**^			
IIIC/D + M1a + M1b	16/33	1	
M1c + M1d	31/37	3.84 (2.06–7.17)	< 0.0001
**Gender**			
Female	12/25	1	
Male	35/45	2.47 (1.28–4.80)	0.007
**Age, years**			
≤ 60	28/37	1	
> 60	19/33	0.64 (0.35–1.15)	0.136
**ECOG PS**^**f**^			
0	20/38	1	
≥ 1	27/32	2.91 (1.62–5.26)	< 0.0001

^a^Event: death

^b^HR, Hazard Ratio; CI Confidence Interval; *P*, probability. Estimated by Cox's regression model

^c^Group: (a) osteopontin ≤ 33,687.82 pg/ml and IL-8 ≤ 4.32 pg/ml; group (b): osteopontin > 33,687.82 pg/ml and IL-8 ≤ 4.32 pg/ml; group (c): osteopontin ≤ 33,687.82 pg/ml and IL-8 > 4.32 pg/ml; group (d): osteopontin > 33,687.82 pg/ml and IL-8 > 4.32 pg/ml

^d^ > 1.5 upper limit of normal values

^e^Disease stage before the beginning of targeted therapy according to 8th Edition of the AJCC *Cancer Staging Manual*

^f^*ECOG PS *Eastern Cooperative Oncology Group Performance Status

Among our cohort of patients, 23 Rs changed therapy after experiencing progression. They were treated with ipilimumab (7 cases), nivolumab (7 cases), pembrolizumab (8 cases) or the combination of nivolumab and ipilimumab (1 case). We considered the possibility that the estimation of OS of patients treated with targeted therapy and its association with baseline levels of IL-8 and osteopontin could be affected by the inclusion of patients who had received immunotherapy after progression, thus achieving a possible improvement in survival. Kaplan–Meier and univariate Cox regression analyses were, therefore, performed also after excluding from the study population the 23 patients who had received second-line therapy.

**Fig. 5:**
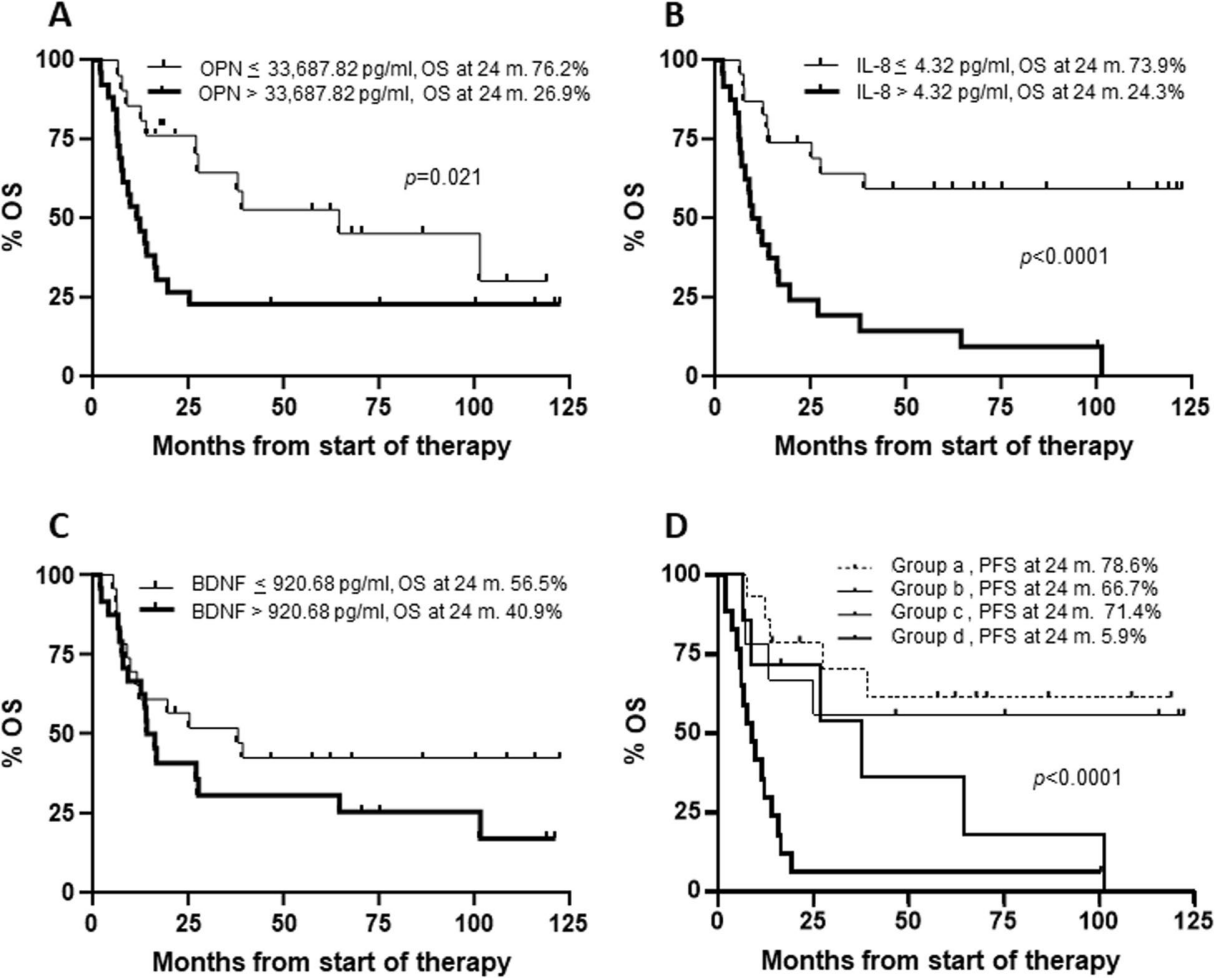
Kaplan–Meier curves for overall survival (OS) of melanoma patients treated with targeted therapy after exclusion of those receiving immunotherapy at disease progression. Patients (n. 47) were categorized based on their T0 levels of osteopontin (**A**), IL-8 (**B**), BDNF(**C**), osteopontin and IL-8 combinations (**D**). Group (a): osteopontin ≤ 33,687.82 pg/ml and IL-8 ≤ 4.32 pg/ml; group (b): osteopontin > 33,687.82 pg/ml and IL-8 ≤ 4.32 pg/ml; group (c): osteopontin ≤ 33,687.82 pg/ml and IL-8 > 4.32 pg/ml; group (d): osteopontin > 33,687.82 pg/ml and IL-8 > 4.32 pg/ml. *p*-values are from log-rank test (**A**, **B**, **C**) and log-rank test for trend (**D**). The estimated percentages of OS at 24 months from the start of therapy are also reported for each comparison

Median OS for the remaining 47 patients was 19.4 months. Median OS was 11.8 and 64.5 months for patients with high and low osteopontin, respectively (*p* = 0.021), and 10.6 months for patients with high IL-8 *vs* not reached for those with low IL-8 (*p* < 0.0001) (Fig. 5A and B). Patients with elevated levels of BDNF exhibited a worse median OS than those with low levels of the cytokine (15.1 *v*s 37.9 months), although the difference was not statistically significant (Fig. 4C). Median OS was not reached for patients of group (a) and (b), and 37.9 and 9.1 months for patients of group (c) and (d), respectively (log-rank test for trend, *p* < 0.0001) (Fig. 5D). Univariate analysis conducted for osteopontin, IL-8 and their combinations, confirmed a significantly increased risk of mortality for patients with high baseline levels of osteopontin, IL-8 and the (d) combination of these cytokines (Table 5).

**Table 5:** Univariate Cox regression analysis for mortality according to T0 plasma levels of osteopontin, IL-8 and clinical features of patients not subjected to second-line therapy

Characteristics	Events^a^/Patients	HR^b^ (95% CI^b^)	*P*^b^
**Osteopontin pg/ml**			
≤ 33,687.82	11/21	1	
> 33,687.82	20/26	2.34 (1.12–4.92)	0.024
**IL-8 pg/ml**			
≤ 4.32	9/23	1	
> 4.32	22/24	4.47 (2.02–9.87)	< 0.0001
**Osteopontin and IL-8 combinations**^**c**^			
Group a	5/14	1	
Group b + group c	10/16	1.90 (0.65–5.60)	0.244
Group d	16/17	7.35 (2.59–20.85)	< 0.0001
**sLDH**			
Normal	14/28	1	
High^d^	17/19	7.14 (2.90–17.61)	< 0.0001
**Stage**^**e**^			
IIIC/D + M1a + M1b	9/19	1	
M1c + M1d	22/28	3.27 (1.48–7.23)	0.003
**Gender**			
Female	8/17	1	
Male	23/30	2.35 (1.04–5.28)	0.039
**Age, years**			
≤ 60	17/23	1	
> 60	14/24	0.72 (0.35–1.47)	0.366
**ECOG PS**^**f**^			
0	13/25	1	
≥ 1	18/22	2.97 (1.43–6.19)	0.004

^a^Event: death

^b^*HR *Hazard Ratio, *CI *Confidence Interval, *P *probability. Estimated by Cox's regression model

^c^Group: (a) osteopontin ≤ 33,687.82 pg/ml and IL-8 ≤ 4.32 pg/ml; group (b): osteopontin > 33,687.82 pg/ml and IL-8 ≤ 4.32 pg/ml; group (c): osteopontin ≤ 33,687.82 pg/ml and IL-8 > 4.32 pg/ml; group (d): osteopontin > 33,687.82 pg/ml and IL-8 > 4.32 pg/ml

^d^ > 1.5 upper limit of normal values

^e^Disease stage before the beginning of targeted therapy according to 8th Edition of the AJCC *Cancer Staging Manual*

^f^*ECOG PS *Eastern Cooperative Oncology Group Performance Status

### Multivariate COX regression analysis of the association of osteopontin and IL-8 T0 plasma levels with PFS ad OS

To assess whether T0 levels of osteopontin, IL-8 or their combinations were independent prognostic factors for PFS and/or OS, multivariate Cox regression analyses were performed. Age was not included in the Cox models because not significantly associated with PFS and OS in univariate analyses. Additionally, since stage and LDH resulted strongly associated (Fisher’s exact test p < 0.0001), separate Cox models including either LDH or stage were carried out to avoid multicollinearity.

After adjusting for sex, ECOG PS and LDH, T0 levels of osteopontin, IL-8 and their combinations did not demonstrate independent associations with PFS, whereas high IL-8 and the (d) combination of IL-8 and osteopontin remained independent risk factors for disease progression when stage replaced LDH in the Cox models (Table 6). On the other hand, after adjusting for sex, ECOG PS and either LDH or stage, high osteopontin, high IL-8 and the (b + c) and (d) combinations of the two cytokines resulted independently associated with a significantly increased risk of mortality compared to low osteopontin, low IL-8 and the (a) cytokine combination, respectively (Table 7).

**Table 6:** Multivariate Cox regression analysis for disease progression according to T0 plasma levels of osteopontin, IL-8 and clinical features of all patients

Characteristics	HR^a^ (95% CI^a^)	*P*^a^
*model 1*^*b*^		
**Osteopontin**		
≤ 33,687.82	1	
> 33,687.82	1.35 (0.78–2.33)	0.282
*model 2*^*c*^		
**Osteopontin**		
≤ 33,687.82	1	
> 33,687.82	1.42 (0.83–2.43)	0.205
*model 3*^*d*^		
**IL-8**		
≤ 4.32	1	
> 4.32	1.76 (0.97–3.20)	0.064
*model 4*^*e*^		
**IL-8**		
≤ 4.32	1	
> 4.32	2.50 (1.41–4.41)	0.002
*model 5*^*f*^		
**Osteopontin and IL-8 combinations**^**h**^		
Group a	1	
Group b + group c	1.72 (0.83–3.56)	0.141
Group d	1.92 (0.91–4.07)	0.088
*model 6*^*g*^		
**Osteopontin and IL-8 combinations**^**h**^		
Group a	1	
Group b + group c	1.74 (0.84–3.60)	0.133
Group d	2.77 (1.34–5.71)	0.006

^a^*HR *Hazard Ratio, *CI *Confidence Interval, *P* probability. Estimated by Cox's regression model

^b^variables included in model 1 are osteopontin, sex, ECOG PS and LDH

^c^variables included in model 2 are osteopontin, sex, ECOG PS and stage

^d^variables included in model 3 are IL-8, sex, ECOG PS and LDH

^e^variables included in model 4 are IL-8, sex, ECOG PS and stage

^f^variables included in model 5 are osteopontin and IL-8 combinations, sex, ECOG PS and LDH

^g^variables included in model 6 are osteopontin and IL-8 combinations, sex, ECOG PS and stage

^h^Groups: (a) osteopontin ≤ 33,687.82 pg/ml and IL-8 ≤ 4.32 pg/ml; group (b): osteopontin > 33,687.82 pg/ml and IL-8 ≤ 4.32 pg/ml; group (c): osteopontin ≤ 33,687.82 pg/ml and IL-8 > 4.32 pg/ml; group (d): osteopontin > 33,687.82 pg/ml and IL-8 > 4.32 pg/ml

**Table 7:** Multivariate Cox regression analysis for mortality according to T0 plasma levels of osteopontin, IL-8 and clinical features of patients

	**70 patients**		**47 patients**	
**Characteristics**	**HR**^**a**^** (95% CI**^**a**^**)**	***P***^**a**^	**HR**^**a**^** (95% CI**^**a**^**)**	***P***^**a**^
*model 1*^*b*^				
**Osteopontin**				
≤ 33,687.82	1		1	
> 33,687.82	1.95 (1.05–3.61)	0.034	1.69 (0.77–3.70)	0.19
*model 2*^*c*^				
**Osteopontin**				
≤ 33,687.82	1		1	
> 33,687.82	2.09 (1.14–3.82)	0.017	1. 98 (0.92–4.29)	0.08
*model 3*^*d*^				
**IL-8**				
≤ 4.32	1		1	
> 4.32	3.12 (1.57–6.23)	0.001	3.52 (1.51–8.21)	0.004
*model 4*^*e*^				
**IL-8**				
≤ 4.32	1		1	
> 4.32	4.18 (2.14–8.19)	< 0.0001	4.35 (1.98–10.02)	0.004
*model 5*^*f*^				
**Osteopontin and IL-8 combinations**^**h**^				
Group a	1		1	
Group b + group c	3.76 (1.49–9.46)	0.005	3.55 (1.07–11.83)	0.039
Group d	4.39 (1.70–11.33)	0.002	3.73 (1.20–11.61)	0.023
*model 6*^*g*^				
**Osteopontin and IL-8 combinations**^**h**^				
Group a	1		1	
Group b + group c	3.98 (1.57–10.14)	0.004	2.59 (0.80–8.37)	0.111
Group d	6.05 (2.42–15.14)	< 0.0001	5.62 (1.89–16.74)	0.002

^a^HR, Hazard Ratio; CI, Confidence Interval; P probability. Estimated by Cox's regression model

^b^variables included in model 1 are osteopontin, sex, ECOG PS and LDH

^c^variables included in model 2 are osteopontin, sex, ECOG PS and stage

^d^variables included in model 3 are IL-8, sex, ECOG PS and LDH

^e^variables included in model 4 are IL-8, sex, ECOGPS and stage

^f^variables included in model 5 are osteopontin and IL-8 combinations, sex, ECOG PS and LDH

^g^variables included in model 6 are osteopontin and IL-8 combinations, sex, ECOG PS and stage

^h^Groups: (a) osteopontin ≤ 33,687.82 pg/ml and IL-8 ≤ 4.32 pg/ml; group (b): osteopontin > 33,687.82 pg/ml and IL-8 ≤ 4.32 pg/ml; group (c): osteopontin ≤ 33,687.82 pg/ml and IL-8 > 4.32 pg/ml; group (d): osteopontin > 33,687.82 pg/ml and IL-8 > 4.32 pg/ml

After excluding from the analysis patients who had received immunotherapy after progression on BRAFi and MEKi, high IL-8 and the (d) combination of IL-8 and osteopontin, remained independently associated with a significantly higher risk of mortality in all Cox models. Patients with high osteopontin and those included in group (b + c) also reported an increased risk of mortality, but the statistical significance was achieved only for group (b + c) in the Cox model including LDH (Table 7).

## Discussion

Currently, LDH stands as the sole circulating biomarker of prognostic significance incorporated into melanoma staging by the American Joint Committee on Cancer (AJCC) [57]. Nonetheless, specific circulating cytokines or cytokine signatures are emerging as promising non-invasive biomarkers for melanoma diagnosis and/or assessment of patient prognosis and response to therapy [17, 18, 23, 35].

In the present study, we demonstrate that baseline circulating levels of IL-8 and osteopontin may serve as valuable biomarkers for predicting response to treatment and/or prognosis in melanoma patients undergoing targeted therapy.

IL-8, also known as CXCL8, is a pleiotropic chemokine produced by numerous normal cell types, including those of the immune system, as well as by fibroblasts, epithelial and endothelial cells. Moreover, it is over-expressed by a variety of cancers [58, 59]. IL-8 binds to CXCR1 and CXCR2 receptors—expressed by immune and endothelial cells, and by most cancer cell types—activating different downstream signaling pathways, including PI3K/AKT, MAPK, and PKC [58, 59]. IL-8 exerts a pro-tumorigenic activity, by stimulating angiogenesis, recruiting immune suppressive cells into the tumor microenvironment, and promoting epithelial-mesenchymal transition, proliferation and therapy resistance of cancer cells [58, 59].

In melanoma lesions, the expression of IL-8 and its receptors has been shown to correlate with tumor progression [60, 61]. Accordingly, studies in vitro and in animal models have demonstrated that the IL-8/CXCR1/2 axis can support melanoma growth and metastasis, both directly, sustaining autocrine loops of proliferation and invasiveness, and indirectly, promoting angiogenesis and immunosuppression [60, 61].

Several investigations have reported significantly higher levels of IL-8 in serum/plasma of patients with primary or metastatic melanoma compared with healthy controls, and have shown a positive correlation of IL-8 levels with either Breslow thickness, or with disease stage, or tumor burden or patient worse OS [60–63]. Circulating level of IL-8 has also been shown to represent a biomarker of clinical outcomes in melanoma patients treated with immunotherapy or targeted therapy. Regarding immunotherapy, Sanmamed et al. [43] showed that a decrease in the level of serum IL-8 after 12–16 weeks of therapy was linked to objective responses in patients treated with ipilimumab, whereas Jamal et al. [37] demonstrated that high baseline plasma levels of IL-8 were associated with unresponsiveness to therapy and worse OS in patients treated with carboplatin, paclitaxel and ipilimumab. In patients receiving anti-PD-1 therapy or nivolumab plus ipilimumab, early changes in serum IL-8 could predict clinical outcomes, with a decrease of the cytokine levels associated with responsiveness to therapy and longer OS [38]. Worse OS associated with elevated pre-therapy levels of serum IL-8 was also demonstrated by Schalper et al. [40] in a study involving 1,344 patients with different type of cancer treated with nivolumab or nivolumab plus ipilimumab, among which 887 had melanoma.

Only limited data are available on the potential role of circulating IL-8 as biomarker of response or survival in patients treated with BRAFi and MEKi. In a study involving 24 patients receiving BRAFi alone or in combination with MEKi, Wilmott et al. [42] demonstrated that IL-8 serum concentration determined after 3–15 days of therapy was significantly lower than that measured at baseline, and that a greater fold reduction correlated with a worse OS. However, no association was found between baseline IL-8 levels and RECIST response, PFS and OS. In an additional investigation, Sanmamed et al. [43] demonstrated that in 16 melanoma patients treated with BRAFi monotherapy, serum levels of IL-8 at the time of best response were significantly lower than those detected at baseline and raised again at progression on treatment. Moreover, patients with high baseline concentrations of IL-8 exhibited worse OS.

In our cohort of patients, baseline levels of IL-8 resulted strongly associated with PFS and OS. Kaplan–Meier curves revealed that patients with IL-8 T0 levels > 4.32 pg/ml had poorer PFS and OS compared to those with IL-8 T0 levels ≤ 4.32 pg/ml, while univariate Cox regression analysis evidenced a significantly higher risk of short-term progression and mortality. The association of high pre-therapy levels of IL-8 with worse OS persisted even after excluding from the analysis patients who had received immunotherapy after progression. These results, obtained in cohort of patients larger than that studied by Sanmamed et al. [43], confirm the negative prognostic value for OS of high baseline levels of IL-8 in patients undergoing targeted therapy. They are also consistent with the findings reported by Jamal et al. [37] and Schalper et al. [40] for melanoma patients receiving immunotherapy. Importantly, unlike previous investigations in melanoma patients treated with BRAFi and MEKi, we conducted a multivariate analysis to assess the independence of IL-8 baseline levels from other well recognized prognostic factors for PFS and OS. This analysis demonstrated that high circulating IL-8 remained an independent risk factor for disease progression after adjusting for sex, ECOG PS and stage, and for mortality after adjusting for sex, ECOG PS and either LDH or stage. These results further highlight the potential utility of circulating IL-8 to refine prognosis evaluation of patients undergoing targeted therapy. Unfortunately, baseline concentration of IL-8 did not result a useful biomarker to identify patients more likely to respond to therapy. Indeed, even though the median value of plasma IL-8 was higher in the group of NRs as compared with the group of Rs, the difference did not reach the statistical significance. Moreover, ROC analysis showed a poor ability of this cytokine to discriminate between Rs and NRs.

Regarding IL-8 variation during therapy, no significant changes from T0 to T2 were detected in NRs, in line with previous findings in melanoma patients treated with immunotherapy [38, 43]. In contrast, we observed a significant reduction of circulating IL-8 from T0 to T2 and a subsequent rise of the cytokine at TP in the cohort of 39 Rs for which all the three matched sample were available. This finding is consistent with the studies of Wilmott et al. [42] and Sanmamed et al. [43], and also with previous investigations on melanoma specimens and cell lines. Indeed, in patients treated with vemurafenib or the combination of dabrafenib + trametinib, a significant decrease of IL-8 mRNA was observed in tumor biopsies collected 10–14 day after the start of therapy with respect to those obtained at baseline [64], while a strong inhibition of IL-8 secretion was evidenced in human melanoma cell lines treated in vitro with either vemurafenib or tramentinib [65]. However, the decrease of circulating IL-8 from T0 to T2 was not evidenced when all Rs (i.e. 60 patients) were considered. This discrepancy can be explain taking into account that the sub-cohort of 39 Rs was more homogenous than that of 60 Rs, comprising only patients who had progressed on therapy. As it could be expected, this sub-cohort included a higher proportion of patients with IL-8 T0 concentration above 4.14 pg/ml (i.e. the median value of entire cohort of Rs) thus making it possible to highlight a decrease in the cytokine median concentration from T0 to T2.

Osteopontin, or secreted phosphoprotein 1 (SPP1) is a multifunctional protein expressed by various normal cell types, including those of the immune system, osteoblasts, osteoclasts, fibroblasts, epithelial cells, nerve cells and endothelial cells [49, 56, 66]. Osteopontin interacts with CD44 and integrin family receptors and with inducibile T-cell costimulatory ligand [49, 56, 66, 67], activating several intracellular signaling pathways, including PI3K/AKT, MAPK, JAK/STAT and Wnt/β-catenin [56]. Osteopontin is involved in numerous physiological processes, such as bone remodeling, innate and adaptive immune responses, wound healing and angiogenesis [49, 66].

Dysregulated expression of osteopontin has been implicated in autoimmune diseases, atherosclerosis, psoriasis and cancer [49, 56, 66, 68]. The oncogenic activity of osteopontin has been linked to its ability to promote cancer cell proliferation, survival and invasiveness, as well as tumor angiogenesis, and an immunosuppressive tumor microenvironment [49, 50, 56, 66, 69]. Aberrant expression of osteopontin has also been implicated in tumor drug resistance in a variety of cancers [51, 56, 70].

In melanoma, the tumor-promoting function of osteopontin has been demonstrate by several functional studies performed in vitro on human and murine tumor cells and in vivo in animal models [49, 50]. Moreover, osteopontin was consistently found to be overexpressed in primary and metastatic melanomas as compared with benign nevi [30, 71–75] or normal skin [76, 77]. However, comparing metastases and primary tumors, several investigations [75–78], but not all [30, 71, 72], evidenced higher osteopontin levels in metastases. Similarly, while some studies failed to identify a correlation between osteopontin expression levels and clinicopathological features of primary tumors and/or patient survival outcomes [30, 71, 72], other investigations clearly demonstrated the association of elevated osteopontin levels with increasing Breslow thickness and mitotic index, as well as with other unfavorable histological parameters, such as nodular subtype and presence of ulceration [77–80], or poorer recurrence-free survival and/or OS [77–81].

Circulating levels of osteopontin were found to be higher in melanoma patients as compared with healthy subjects [31–33], higher in patients with metastatic disease as compared with non-metastatic patients, and positively associated with Breslow thickness [29, 30]. Furthermore, osteopontin levels were found to be more elevated in patients with sentinel lymph node metastases than in those with negative sentinel lymph nodes [32, 33]. Overall, those investigations highlight the negative prognostic value of high circulating level of osteopontin in melanoma patients, in line with the oncogenic activity showed for this cytokine in functional studies.

To the best of our knowledge, the possible association of circulating osteopontin with clinical outcomes in melanoma patients undergoing targeted therapy or immunotherapy has not yet been investigated. Our results show for the first time that osteopontin could serve as a non-invasive and useful biomarker for predicting clinical response and prognosis in patients treated with BRAFi and MEKi. Indeed, pre-therapy levels of osteopontin were significantly higher in the group of NRs as compared with the group of Rs, decreased only in the latter group after two months of therapy, rising again upon the development of secondary resistance to treatment, i.e. at progression. Additionally, the baseline level of osteopontin showed promising discriminatory ability between Rs and NRs. Moreover, although the statistical significance was not reached, a clear trend toward a worse PFS was evidenced for patients with high baseline levels of osteopontin. Indeed, only 24.5% of those patients was free of progression after 24 months of therapy, a percentage only marginally higher than those observed for patients with elevated circulating IL-8 (i.e. 21.3%). Notably, similar to IL-8, patients with elevated osteopontin levels exhibited significantly lower OS and higher risk of mortality compared to those with lower levels of the cytokine. Finally, even after adjusting for sex, ECOG PS and either LDH or stage in multivariate analysis, patients with baseline osteopontin > 33,687.82 pg/ml continued to show an increased risk of mortality, although statistical significance was not achieved after excluding patients who received post-progression immunotherapy, likely due to the reduced size of the cohort.

Overall, our findings align with the oncogenic role of osteopontin in melanoma, as supported by existing literature [49, 50] and are consistent with previous observations in patients with different tumors undergoing chemotherapy or immunotherapy. For instance, a high baseline level of circulating osteopontin was a predictor of poor clinical response and inferior PFS and/or OS in patients with nasopharyngeal cancer subjected to radiotherapy or concurrent chemoradiation [82], in non-small-cell lung cancer patients receiving platinum-based chemotherapy or nivolumab [83, 84], as well as in locally advanced or metastatic breast cancer patients treated with neoadjuvant chemotherapy [85] or docetaxel plus cisplatin or carboplatin [86], respectively.

The observation that elevated baseline concentrations of either IL-8 or osteopontin were negative prognostic factors for PFS and OS, prompted us to investigate survival outcomes considering the levels of both cytokines in each individual. As expected, Kaplan–Meier curves showed that the median PFS and OS of patients with elevate levels of both IL-8 and osteopontin were lower than those of patients with low levels of both cytokines and patients with only high IL-8 or osteopontin. Crucially, even after adjusting for sex ECOG PS and either LDH or stage, a significantly increased risk of mortality persisted for patients with unfavorable levels of both cytokines. These findings highlight the potential of concurrent assessment of baseline circulating levels of IL-8 and osteopontin to improve prognostication of survival outcomes in patients undergoing treatment with BRAFi and MEKi. Moreover, they suggest that targeting IL-8 and osteopontin or their receptors may provide new avenues to improve clinical efficacy of targeted therapy. Notably, inhibitors of CXCR1/2 (SX-682) or CXCR2 (AZD5069, MK-7123), as well as a fully human monoclonal antibody targeting IL-8 (BSM-986253), are already under clinical investigation in solid tumors in combination with immune checkpoint inhibitors (https://clinicaltrials.gov).

BDNF is a growth factor belonging to the family of neutrophins, which are involved in the regulation of neuronal differentiation, survival, functions, and plasticity [87, 88]. BDNF is primarily expressed in the brain, but is also produced by different type of normal cells, such as platelet, lymphocytes, monocytes, vascular smooth muscle, and cardiomyocytes [88]. BDNF binds to the tropomyosin-related kinase B (TrkB) receptor and the neutrophin receptor P75^NTR^ and activates multiple signaling pathways, including RAS/MAPK, PI3K/AKT, PLC-γ/PKC, JAK/STAT [87, 88].

Overexpression of BDNF and/or TrkB has been observed in a variety of tumors, including neuroblastoma, lung, breast, stomach, colorectal and head and neck cancers, and shown to promote tumor cell proliferation, survival, epithelial-mesenchymal transition, invasiveness, and drug resistance as well as angiogenesis [89–93]. Accordingly, elevated expression of BDNF/TrkB in neoplastic tissue has been linked to poor patients’ prognosis in several cancers [89–94].

The limited number of studies evaluating BDNF expression in serum of cancer patients have produced diverse results. With respect to samples obtained from healthy controls, either decreased [95, 96] or increased [97–100] levels of that cytokine have been documented in cancer patients, depending on the tumor type investigated. In cancer patients undergoing systemic therapy, elevated baseline levels of BDNF have been associated with clinical response and improved OS in patients with multiple myeloma treated with bortezomib, and/or thalidomide-based chemotherapy [53], but with poorer PFS in patients with pancreatic adenocarcinoma receiving FOLFIRINOX neoadjuvant chemotherapy regimen [101]. Furthermore, an increase of BDNF after 2–3 weeks of therapy, but not the baseline level, correlated with response to therapy and better PFS in gastric cancer patients treated with immunotherapy [52].

Expression of BDNF has been documented in benign pigment cell lesions and melanomas but not in normal skin [45, 48]. In melanoma, BDNF positivity was found to be higher in metastases than in primary tumors [45, 48], where it resulted associated with the presence of ulceration and higher Clark level, Breslow thickness and disease stage [48]. Moreover, a poorer relapse-free survival and OS was observed for patients with BDNF-positive tumors as compared with those with BDNF-negative tumors [48]. Importantly, concomitant TrkB and BDNF expression has been demonstrated in melanoma specimens [45, 48], suggesting that the BDNF/TrkB pathway can support an autocrine loop of growth and progression in this tumor. Surprisingly, BDNF serum concentration was found to be lower in melanoma patients than in healthy subjects, with no difference between primary or metastatic disease [36]. In patients with primary tumors, BDNF serum level was inversely correlated with Breslow thickness but not associated with OS [36].

In our current study, we show that BDNF was highly expressed in plasma of metastatic melanoma patients undergoing targeted therapy, with no significant differences between Rs and NRs across the three time points analyzed. However, we observed a clear trend toward a poorer OS in patients with high BDNF plasma concentration. Indeed, although the statistical significance was not reached, patients with baseline BDNF levels ≤ 920.68 pg/ml demonstrated a median OS more than twice as long as patients with BDNF levels exceeding this threshold, either considering the entire cohort of patients or the sub-cohort in which patients receiving immunotherapy at progression were excluded. This finding suggests that although an elevated level of circulating BDNF does not influence clinical response to targeted therapy, it can adversely affect OS, in line with the negative prognostic value demonstrated for high BDNF expression in melanoma specimens [48]. However, further studies in a larger cohort of patients are required to better define the potential of circulating BDNF as biomarkers of OS in melanoma patients undergoing targeted therapy.

IL-8, osteopontin and BDNF can be produced by melanoma cells and various type of tumor-associated stromal cells and can act in paracrine and autocrine fashion, promoting cancer cell proliferation and invasiveness, an immunosuppressive microenvironment and angiogenesis. In this study, we did not determine the contribution of the different components of tumor tissue to the cytokine circulating levels. However, regardless of the actual source of the investigated cytokines in patients’ plasma, our findings demonstrate that determination of pre-therapy levels of IL-8 and osteopontin could aid prognosis evaluation in patients undergoing targeted therapy.

Our study provides clinically relevant results, particularly considering the long median follow-up of patients. However, it has some limitations that warrant consideration. It was retrospective in nature and included a relatively small number of NRs. Furthermore, we could not separately analyze patients who received BRAFi + MEKi combination therapy and those who were treated with BRAFi alone, due to the limited number of the latter patients. Although our findings are promising, they need to be validated in a larger prospective study.

## Conclusions

Our study reveals that high pre-therapy plasma levels of IL-8 were associated with significantly shorter PFS and OS and higher risk of progression and mortality in melanoma patients undergoing targeted therapy. Importantly, an elevated IL-8 baseline level remained an independent adverse prognostic factor for OS after adjusting for sex, ECOG PS, and either LDH—the sole circulating biomarker included in melanoma staging by the AJCC—or stage. Pre-therapy levels of osteopontin were significantly higher in NRs as compared with Rs and an elevated osteopontin concentration was significantly associated with worse OS and increased risk of death. Crucially, patients exhibiting high levels of both IL-8 and osteopontin experienced the poorest outcomes in terms of PFS and OS. Even after accounting for sex, ECOG PS, and either LDH or stage in multivariate analysis, this cytokine combination remained independently associated with a three- to six-fold increased risk of mortality.

Adding assessment of pre-therapy IL-8 and osteopontin levels to currently recognized prognostic factors, could refine prognosis evaluation of patients undergoing targeted therapy. Moreover, IL-8 and osteopontin deserve attention as potential therapeutic targets to improve clinical efficacy of BRAFi and MEKi.

### Supplementary Information

Additional file 1. Supplementary Fig. 1. Box-and-whisker diagrams of cytokine T0 plasma levels in melanoma patients grouped according to disease stage. Osteopontin (OPN) (A), IL-8 (B) and BDNF (C) levels were measured by xMAP technology in plasma samples obtained from 70 patients before treatment initiation. The edges of each box represent the 75th and 25th percentile, respectively, and whiskers are defined according to Tukey method. The horizontal bar within each box indicates the median. The outliers are reported as dots. Data were analyzed by nonparametric Kruskal–Wallis test followed by the post-hoc Dunn’s test for multiple comparisons. Panel (A): *p = 0.013 for comparison between M1c and IIIC/IIID; #p = 0.031 for comparison between M1c and M1d. Panel (B): ##p = 0.005 for comparison between M1c and M1b

## Data Availability

All data supporting the findings of this study are available within the article and its supplementary information and from the corresponding author upon reasonable request.

## Abbreviations

AJCC: American Joint Committee on Cancer
AUC: Area Under Curve
BRAFi: BRAF inhibitors
BDNF: Brain Derived Neurotrophic Factor
CCL4: C-C motif chemokine ligand 4
CI: Confidential Interval
CR: Complete Response
CT: Computer Tomography
CTLA-4: cytotoxic T-lymphocyte associated protein 4
CXCL8: C-X-C motif chemokine ligand 8
CXCR: C-X-C motif chemokine receptor
ECOG: Eastern Cooperative Oncology Group
HR: Hazard Ratio
IL-8: Interleukin-8
IQR: Interquartile Range
LAG-3: Lymphocyte activation gene 3
LDH: Lactate Dehydrogenase
LIF: Leukemia Inhibitory Factor
MEKi: MEK inhibitors
NRs: Non-Responders
ORR: Objective Response Rate
OS: Overall Survival
PD: Progressive Disease
PD-1: Programmed cell death protein 1
PFS: Progression-Free Survival
PD-L1: Programmed cell death-ligand 1
PR: Partial Response
PS: Performance Status
SPP1: Secreted Phosphoprotein 1
ROC: Receiver operating characteristics
SD: Stable Disease
Rs: Responders
TrkB: Tropomyosin-related Kinase B
