# Supplementary figures and images for: Circulating interleukin-8 and osteopontin are promising biomarkers of clinical outcomes in advanced melanoma patients treated with targeted therapy
Source: J Exp Clin Cancer Res. 2024 Aug 15;43:226. doi: 10.1186/s13046-024-03151-3 (PMC11325673; doi:10.1186/s13046-024-03151-3)

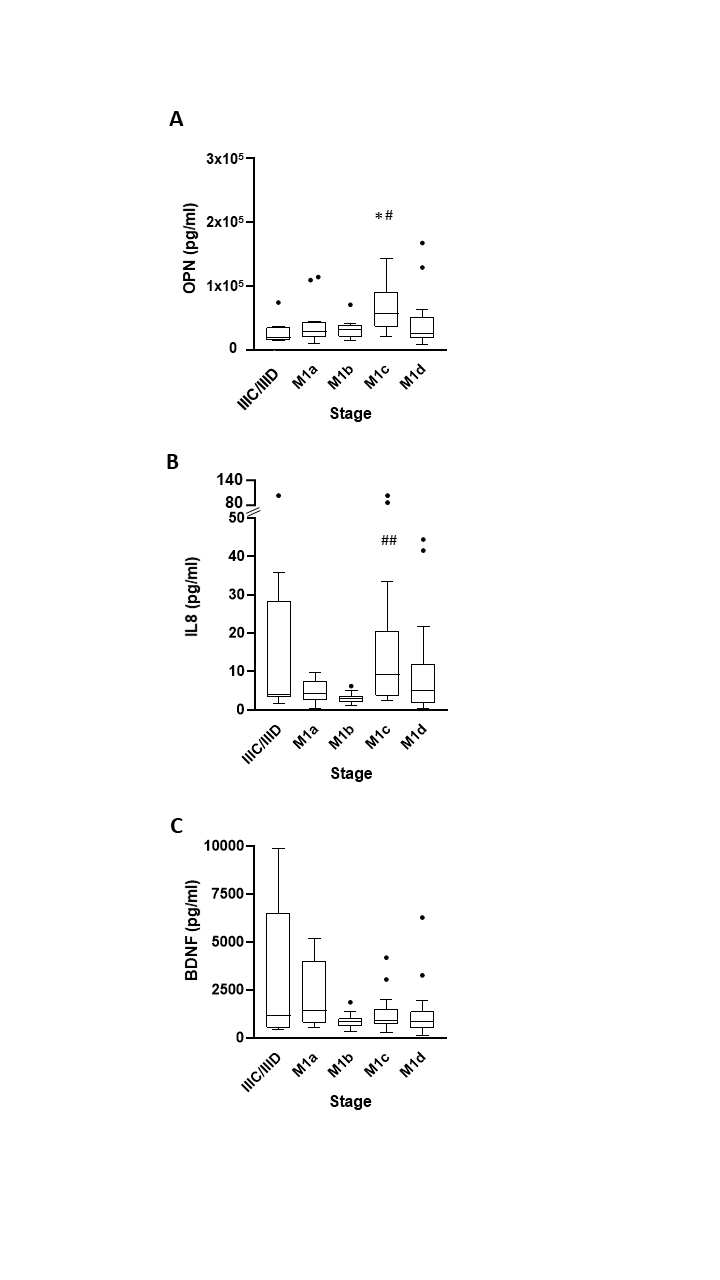

Supplement: Supplementary file 1 — Additional file 1. Supplementary Fig. 1. Box-and-whisker diagrams of cytokine T0 plasma levels in melanoma patients grouped according to disease stage. Osteopontin (OPN) (A), IL-8 (B) and BDNF (C) levels were measured by xMAP technology in plasma samples obtained from 70 patients before treatment initiation. The edges of each box represent the 75th and 25th percentile, respectively, and whiskers are defined according to Tukey method. The horizontal bar within each box indicates the median. The outliers are reported as dots. Data were analyzed by nonparametric Kruskal–Wallis test followed by the post-hoc Dunn’s test for multiple comparisons. Panel (A): *p = 0.013 for comparison between M1c and IIIC/IIID; #p = 0.031 for comparison between M1c and M1d. Panel (B): ##p = 0.005 for comparison between M1c and M1b [file 13046_2024_3151_MOESM1_ESM.png]
